# New Mono- and Diesters with Imidazoquinolinone Ring- Synthesis, Structure Characterization, and Molecular Modeling

**DOI:** 10.3390/molecules25184303

**Published:** 2020-09-19

**Authors:** Iwona Zarzyka, Antonin Klásek, Karol Hęclik, Antonin Lyčka, Radek Bartošík, Lucjan Dobrowolski

**Affiliations:** 1 Department Organic Chemistry, Institution Rzeszów University of Technology, Powstańców Warszawy 6, 35-959 Rzeszów, Poland; 2 Department Chemistry, Institution Tomas Bata University in Zlin, CZ-76272 Zlin, Czech Republic; klasek@utb.cz (A.K.); ra.bar@seznam.cz (R.B.); 3 Department Biotechnology and Bioinformatic, Institution Rzeszów University of Technology, Powstańców Warszawy 6, 35-959 Rzeszów, Poland; kheclik@prz.edu.pl (K.H.); ldobrowolski@prz.edu.pl (L.D.); 4 Research Institute for Organic Syntheses (VUOS), CZ-53354 Pardubice, Czech Republic; antonin.lycka@uhk.cz

**Keywords:** ammonium thiocyanate, debenzylation, 3-hydroxyquinolinediones, thioxoimidazoquinolinone ring, molecular modeling

## Abstract

The objective of the studies was to synthesize and characterize new mono- and diesters with an imidazoquinolin-2-one ring with the use of 2,3-dihydro-2-thioxo-1H-imidazo[4 ,5-c]-quinolin-4(5*H*)-ones and ethyl bromoacetate. The products were isolated at high yield and characterized by instrumental methods (IR, ^1^H-, ^13^C-, and ^15^N- NMR, MS-ESI, HR-MS, EA). In order to clarify the places of substitution and the structure of the derivatives obtained, molecular modeling of substrates and products was performed. Consideration of the possible tautomeric structures of the substrates confirmed the existence only the most stable keto form. Based on the free energy of monosubstituted ester derivatives, the most stable form were derivatives substituted at sulfur atom of enolic form the used imidazoquinolones. Enolic form referred only to nitrogen atom no 1. The modeling results were consistent with the experimental data. The HOMO electron densities at selected atoms of each substrate has shown that the most reactive atom is sulfur atom. It explained the formation of monoderivatives substituted at sulfur atom. The diester derivatives of the used imidazoquinolones had second substituent at nitrogen atom no. 3. The new diesters can be used as raw material for synthesis of thermally stable polymers, and they can also have biological activity.

## 1. Introduction

Presently, products with high thermal stability are demand by consumer market. Therefore, research trend regarding the synthesis and analysis of the properties the materials which are thermally stable are very popular. Polymers constitute a large group of substances characterized by such properties. There are many different definitions of thermally stable polymers. According to Szczerba, a thermally stable polymer does not degrade during long-term exposure, i.e., during 30,000 h at temperatures up to 170 °C and does not change shape or melt when heated to 400 °C for a short time [1].

In turn, according to Marvel, thermally stable macromolecular compounds during prolonged exposure, i.e., up to 25,000 h at temperatures below 300 °C do not undergo the thermal degradation. Moreover, thermally stable polymer does not change its shape and does not melt during short-term temperature exposure, i.e., up to 300 h at 500 °C [2].

Thermal stability is closely related to the polymer structure. The symmetry of the macromolecule structure, the presence of the side chains and the degree of crosslinking of the polymer also influence significantly the rigidity of macromolecule. It should be emphasized that the presence of side substituents in the main chain or linkers between polymer chains reduce the thermal stability of the polymers [1,3,4,5]. A large part of polymers with increased thermal stability contain aromatic, including heteroaromatic rings in their chains [6]. Polyimides and polyacrylates are the most known groups of thermally stable polymers [7,8]. Studies on new polymers with increased thermal stability refer to the synthesis of new macromolecules or the structure modification of the known polymers with the use of compounds that guarantee the increased thermal stability. Low molecular weight compounds containing an imidazoquinazoline or imidazoquinoline rings are examples of such substances.

In our preceding papers [9,10] we described the preparation and properties of monomers with imidazoquinazoline ring. Diols with imidazoquinazoline ring were used to synthesis of linear polyurethanes. The presence of imidazoquinazoline rings in polyurethane chains makes the polymer is more stable at elevated temperature [11].

**Scheme 1:**
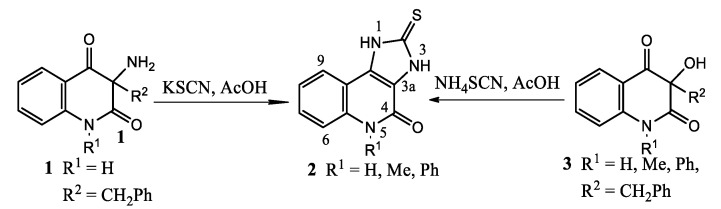
Synthesis scheme of 2,3-dihydro-2-thioxo-1*H*-imidazo[4,5-*c*]quinolin-4(5*H*)-ones (**2**)**.**

Imidazoquinolinones are also heterocyclic compounds and their ring can enhance the thermal stability of polymers. Therefore, we decided to prepare esters with imidazoquinolinone ring. In one of our previous papers [12] we described that the reaction of 1-unsubstituted 3-aminoquinolinediones **1** with potassium thiocyanate in boiling acetic acid yields two structurally diverse products. However, if the starting compounds were substituted with a benzyl group at position 3, C-debenzylation proceeded to give 2,3-dihydro-2-thioxo-1*H*-imidazo[4,5-*c*]quinolin-4(5*H*)-ones **2** as the only reaction products (Scheme 1). The analogous reaction was observed afterwards also with *N*-substituted derivatives [13]. N(5)-substituted compounds **2** were prepared also from 3-hydroxyquinolinediones **3** by the reaction with NH_4_SCN in acetic acid [14].

**Scheme 2:**
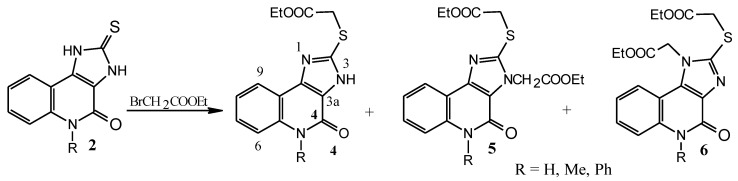
Synthesis scheme of the reaction synthesis of compounds **4**–**6**.

Compound **2**, unsubstituted or substituted at nitrogen atom N(5), where R^1^ is hydrogen, phenyl or methyl group undergoes reaction with ethyl bromoacetate and mono- or disubstituted derivatives are formed (Scheme 2). The synthesis conditions, the structure analysis and characterization of those esters are the subject of this research. The calculations carried out by molecular modeling methods and their results are consistent with the experimental data.

## 2. Results and Discussion

### 2.1. Structure Analysis

Our effort was the preparation of thioxoimidazoquinolones bearing one or two ethoxycarbonylmethyl groups (Scheme 2). Starting compounds **2** were prepared by known procedure from 3-hydroxyderivatives **3** by the reaction with ammonium thiocyanate in acetic acid [15].

The reactions of **2** with ethyl bromoacetate (EBA) were carried out using the molar ratios of **2** to EBA 1:1.1, 1:2.4, and 1:4. The results of these reactions are given in Table 1.

As can be seen from Table 1, the pure dialkylated products **5** arises only at molar ratio 1:4. At lower molar ratios, a considerable quantity of monoalkylated products generate. From Table 1 it can also be seen the high regiospecifity of the reaction. From several possibilities for alkylation compounds **2**, the attack on the enolized C=S group always occurs (compounds **4**). The attack of the second molecule of ethyl bromoacetate on nitrogen atom in position 3 is dominant (compounds **5**), while the formation of compounds **6** (alkylated in positions 1 and 2) occurs only to a small extent (**6a**). Spectral characterization of all obtained products was provided in Table 2. As presumed, the compounds alkylated at two nitrogen atoms and without alkylation at the S-atom were not isolated.

Our attempts on cyclization of **4** with ammonium nitrate [15] were fruitless (only starting material was isolated). Also experiments to convert of **5** to tetracyclic compounds by intramolecular Dieckmann condensation in the presence of basic catalysts (sodium ethoxide, potassium *t*-butoxide, DMAP, and DBU) were unsuccessful. In all cases, only starting material was isolated.

### 2.2. Molecular Modeling

**Scheme 3:**
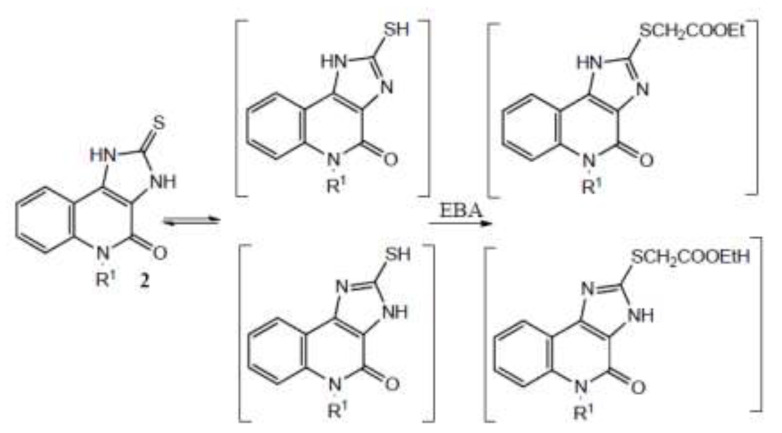
Alkylation of the two possible tautomeric forms of intermediate thiole.

Instrumental analysis of the compounds **2** shows that they exist in keto forms, while the monosubstituted derivatives (**4**) were indicated with a substituent connected to the sulfur atom, so the reaction proceeds with the enol form according Scheme 3.

To clarify the course of the reaction, molecular modeling of the initial compounds were performed and the energies (including Gibbs enthalpy) of possible tautomeric forms of the compounds **2** were calculated.

Compound **2a can** occur in six tautomeric forms: One diketo, three keto-enol and two dienol, while **2b** and **2c** can occur in one diketo and two keto-enol forms what is shown in Table 3.

Based on the calculated values of free energy, an analysis of conformer population was carried out and the percentage shares of tautomeric forms of compounds **2** were calculated, which are listed in the Table 4.

Compound **2**, regardless of the presence and type of the substituent at nitrogen atom no. 5, occurs in 99.99 mol% in the keto form. Since the most stable form of compound **2** is the keto form and the product form structure indicated the reaction of the enol form (N^(1)^-SH) with EBA, it means that the enolization of compound **2** occurs after mixing the reagents just before the reaction with EBA. Such reaction pattern doesn’t depend on the type of the R substituent at nitrogen atom no. 5 (H, Me, Ph).

**Figure 1:**
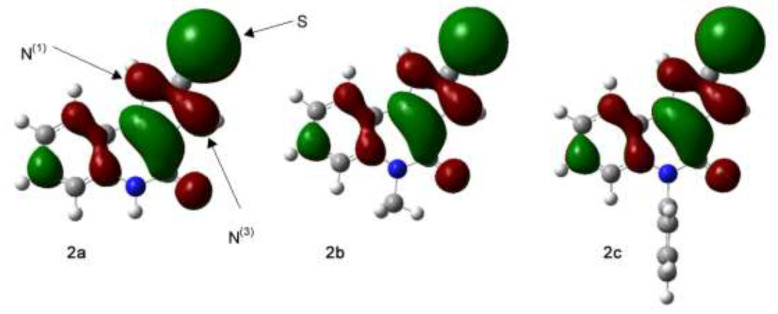
Highest occupied molecular orbitals (HOMO) for compound **2a**, **2b**, **2c.** Isosurface value was set to 0.02.

Considering that the reaction of the compounds **2** with EBA is a nucleophilic substitution, a place of the substitution can be explained with the electron density of the potential nucleophilic reactive places nitrogen atom no. 1 (N^(1)^), nitrogen atom no. 3 (N^(3)^), nitrogen atom no. 5 (N^(5)^), sulfur atom (S), and oxygen atom (O)) in each keto/enol form of the substituted and unsubstituted at nitrogen atom no. 5 compound **2,** see Figure 1.

On this purpose, calculations of HOMO electron densities at selected atoms of molecular orbital [16] were made for all forms of compound **2**. Results shown in Table 5 imply that the highest share (50%) is for the orbital of sulfur atom, and shares of the N^(1)^, N^(3)^, N^(5)^ and O are 5%, 10%, 0%, and 5%, respectively. Thus, in reaction with the EBA, the sulfur atom reacts as nucleophilic agent with the highest probability. It confirms the assumption that enolization occurs just before the reaction of compound **2** with EBA.

In addition, the structure of the set reaction products of compounds **2** with EBA is confirmed by the analysis of conformer population of monosubstituted derivatives of compounds **2**. Based on the calculated Gibbs enthalpy values, shares of monosubstituted derivatives obtained by the reaction of **2** with EBA were calculated and shown in the Table 6. The shares of disubstituted derivatives of compounds **2** are shown in the Table 7. In both tables, last column contains conformer symbol which meaning is explained in Table 8. On the left (right) fragment of arch nitrogen atom No. 1 (3) has its position, while in the middle, sulfur exists. Both nitrogen atoms are marked as circle while sulfur atom is marked as triangle. If the figure (triangle or circle) is solid, it means that substituent chain is placed over the plane of the rings-position below the plane is shown as a clear shape. If the shape (circle/triangle) is below the arch, it means that substituent’s -OC_2_H_5_ fragment is over rings. Outside of the rings position is described with figure position over the arch. The substituent’s chain plane inclination seen from the center of the rings can be to the right or to the left—what is marked as a short line at the right or left side of the figure. Moreover, for 4a compound there is nitrogen atom No. 5, which is described by a circle below the arch and not connected with the arch. It should be mentioned, that there is a few compounds with substituent’s chains in plane of the rings—they are visualized by a line (substituent chain is sticking outwards) or polyline (substituent chain is folded in plane). The conformer structures of compounds 4 and 5 are given in Table 1S and 2S, respectively.

One can see that for the monoderivatives (Table 6), the percentage of compounds substituted at sulfur atom (ideogram: Arch with object (line, polyline, triangle) connected in the middle) is over four times bigger than other substitutions (~81, ~74, and ~67 mol.-% for compound 4a, 4b, and 4c, respectively). Moreover, percentage of compounds substituted at sulfur atom with substituent chain in plane of the rings (ideogram: Arch + line) is in all three cases (4a, 4b, 4c) many times higher than other compounds (~76, ~70, and ~63 mol.-%, respectively). Compounds with the second highest percentage (~7–~10 mol.-%) are these with substituent at nitrogen atom No. 1 (ideogram: Arch + circle connected on the left side). Interesting fact, one can see for the compounds substituted at nitrogen atom No. 3 (ideogram: Arch + circle on the right side): A little percentage for compound 4a and 4b (~2 mol.-%) but three-to-four times bigger percentage for compound 4c (~7 mol.-%), which can be related to the presence of the phenyl ring at nitrogen atom No. 5.

Because of the big value of percentage for compounds substituted in plane of the rings at sulfur atom (Table 6), it is not surprising that all bisderivatives compounds are substituted primarily at the sulfur atom and secondly in another place (nitrogen atom No. 1 or 3). Surprisingly, as it is shown in Table 7, the percentage of all compounds substituted at S and N(1) is extremely low, almost on the verge of error (<0.02 mol.-%).

Percentage of bisderivatives substituted at sulfur atom with substituent chain over the rings (ideogram: Triangle above arch) and nitrogen atom No. 3 with substituent chain (ideogram: Circle at right is out of the arch), in all of three cases (5a, 5b, 5c) is about 30 mol.-%. Worth noting is the fact that this is for only six isomers (three pairs of enantiomers), where the chains of substituents are positioned alternately (over-below the plane of the rings). Each of isomers with the same both substituent’s chains: Over or below the plane, over or outside the rings, and has a mole percentage less than 10 mol.-%.

### 2.3. Thermal Properties

In order to assess a thermal stability of the obtained mono- and diesters with imidazoquinolinone ring, thermogravimetrical analysis was performed.

**Figure 2:**
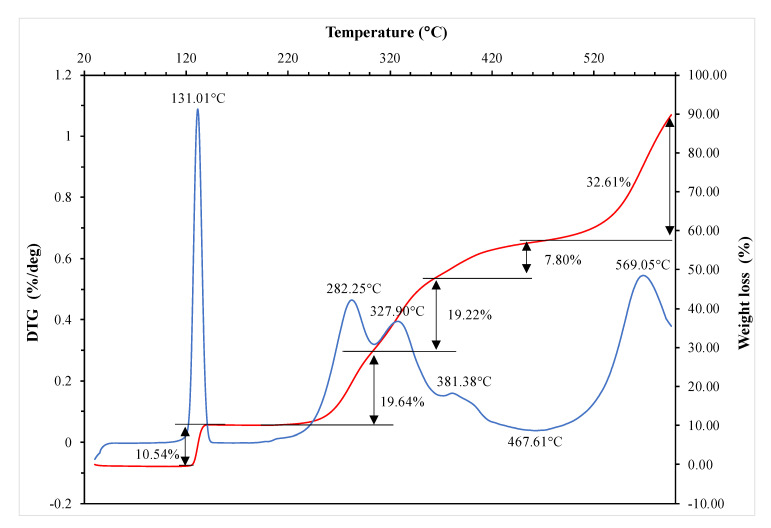
TGA thermogram of diester **5c.**

TG and DTG curves of the diester were shown in Figure 2 on the example of compound **5c**.

Based on thermogram in Figure 2 and the shape of TGA curve and number of peaks on DTG curve, it is seen that decomposition of diester **5c** occurred in about five stages. The first stage of degradation seemed to occur in the temperature range of 120–138 °C and was accompanied by Δm_1_ = 10.54% of mass loss. Nevertheless, this stage did not refer to degradation but it was related to water evaporation. All obtained mono- and diester are highly hygroscopic and sensitive to moisture from the atmosphere. The water presence was confirmed by DSC measurement.

**Figure 3:**
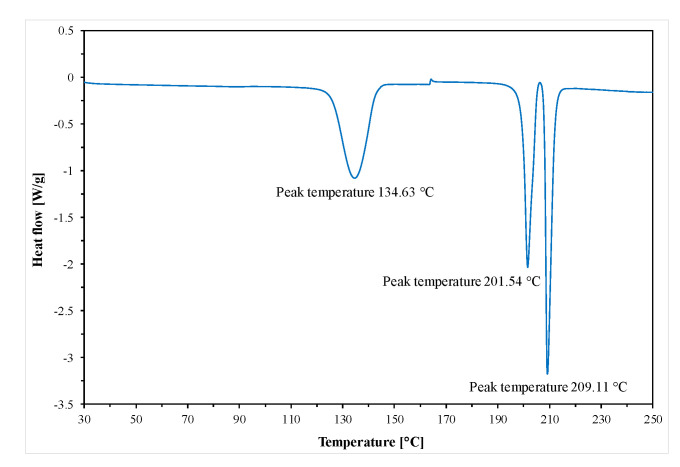
DSC thermogram of diester **5c.**

On DSC thermogram shown in Figure 3, endothermic peak with the peak temperature at 134.63 °C was visible. In second heating, no peak was observed at that temperature range. The peak presence upon first heating and its lack during second heating proved water evaporation [17,18].

The real first stage of diester **5c** degradation appeared in the temperature range 210–305 °C and was accompanied by 19.64% of mass loss. It was connected with one ethoxycarbonylmethylene group degradation.

The second stage was observed in the range of 305–370 °C with a mass loss of 19.22% and indicated degradation of the second ethoxycarbonylmethylene group. Based on the turn of the ethoxycarbonylmethylene group appearance at imidazoquinolinone ring, ester groups degrade in reverse turn. First, the ester group present at nitrogen atom no. 3 undergoes degradation and next that one at sulfur atom. A similar situation took place in the case of hydroxyethyl derivatives of 2H,6H-1-phenylimidazo[1,5-c]quinazoline-3,5-dione [10].

The third stage of degradation was in the range of 370–467 °C and was related to the cleavage of sulfur atom. The temperature of maximum mass loss rate at this stage was 381 °C and the mass loss was Δm_3_ = 7.8%.

The fourth stage of diester **5c** degradation was the last stage. It took place in the range of 467–600 °C and was related to the decomposition of the imidazoquinolinone ring.

The residue at 600 °C of 10.19 wt.-% was probably related to the formation of non-volatile complex structures composed of condensed aromatic rings.

Due to the presence of imidazoquinolone rings, the synthesized esters have good thermal stability, thus they can be used to modification of thermal stability of polymers, similar as other monomers with heterocyclic rings [19,20,21,22,23].

## 3. Materials and Methods

### 3.1. Materials

2-Thioxoimidazoquinolin-2-ones **2** were obtained from 3-hydroxyquinoline-2,4-diones **3** according to procedure [15]. The rest reagents were purchased and used as obtained. *N*,*N*-Dimethylformamide (DMF), pure for analysis, ethyl bromoacetate, pure for analysis, potassium carbonate, pure for analysis, chloroform, pure for analysis, ammonium nitrate, sodium ethoxide, potassium *t*-butoxide, 4-(Dimethylamino)pyridine (DMAP), and 1,8-diazabicyclo[5.4.0]undec-7-ene (DBU) were purchased from Sigma-Aldrich (Darmstadt, Germany).

### 3.2. Syntheses

General Procedure for the Reaction of Compounds 2 with Ethyl Bromoacetate

To the solution of compound 2 (10 mmol) in DMF (40 mL) was added powdered potassium carbonate (40 mmol) and ethyl bromoacetate in quantities given in Table 1. The mixture was stirred at 40 °C for 4 h and extracted with chloroform (4 × 50 mL). Collected extracts were washed with water (3 × 40 mL), dried with anhydrous sodium sulfate and evaporated to dryness. The residue was column chromatographed. Yields of pure product are given in Table 1.

*Ethyl 2-((4-oxo-4,5-dihydro-3H-imidazo[4,5-c]quinolin-2-yl)thio)acetate (***4a***)*. Product characterization: Colorless solid, melting point 218–222 °C (from ethyl acetate). EA: % calcd (found) for C_14_H_13_N_3_O_3_S (303.34): C 55.43 (55.35), H 4.32 (4.10), N 13.85 (13.68), S 10.57 (10.27). IR (KBr): 3338, 3085, 3032, 2979, 2918, 1730, 1649, 1549, 1479, 1462, 1421, 1353, 1334, 1307, 1252, 1201, 1172, 1161, 1110, 1026, 969, 932, 895, 868, 851, 817, 776, 750, 720, 685, 660, 640, 600, 562, 503, 468 cm^−1^. ^1^H, ^13^ C{^1^H}, and ^15^N NMR data are referred in Table 2. EI-MS (*m*/*z*, %) EI-MS: 304.04 (32%), 276.04 (7%), 258.03 (100%), 230.04 (95%), 216.02 (18%), 186.07 (16%). ESI-MS (*m*/*z*): 303 [M + H]^+^, 326 [M + Na]^+^.

*Ethyl 2-((4-oxo-5-methyl-4,5-dihydro-3H-imidazo[4,5-c]-quinolin-2-yl)thio)acetate (***4b***)*. Product characterization: Colorless solid, melting point 231–237 °C (from benzene).EA: % calcd (found) for C_15_H_15_N_3_O_3_S (317.33): C 56.77 (56.71), H 4.76 (4.68), N 13.24 (13.05), S 10.10 (10.03).IR (KBr): 3441, 3083, 2982, 2926, 1730, 1649, 1573, 1481, 1454, 1427, 1404, 1356, 1301, 1256, 1222, 1176, 1164, 1117, 1028, 975, 949, 901, 868, 818, 756, 736, 711, 697, 679, 654, 571 cm^−1^. ^1^H, ^13^ C{^1^H}, and ^15^N NMR data are referred in Table 2. EI-MS (*m*/*z*, %): 318.09 (9%), 290.06 (7%), 272.05 (100%), 244.05 (61%), 230.04 (18%). HR-MS:[M^+^] 318.0905; [M^+^ + 1] 319.0939; [M^+^ + 3] 320.0861.

*Ethyl 2-((4-oxo-5-phenyl-4,5-dihydro-3H-imidazo[4,5-c]-quinolin-2-yl)thio)acetate (***4c***).* Product characterization: Colorless solid, melting point 207–210 °C (from benzene/hexane). EA: % calcd(found) for C_20_H_17_N_3_O_3_S (379.39): C 63.31 (63.51), H 4.52 (4.47), N 11.08 (10.96), S 8.45 (8.41). IR (KBr): 3090, 3036, 2975, 2927, 2753, 1722, 1641, 1571, 1491, 1474, 1448, 1413, 1387, 1353, 1307, 1271, 1225, 1183, 1145, 1095, 1073, 1051, 1024, 975, 924, 900, 861, 842, 814, 760, 710, 678, 659, 575, 555, 520, 448 cm^−1^. ^1^H, ^13^ C{^1^H}, and ^15^N NMR data are referred in Table 2. EI-MS (*m*/*z*, %): 380.11 (7%), 352.07 (6%), 290.06 (7%), 334.06 (82%), 306.07 (100%), 292.05 (57%), 265.04 (37%), 259.07 (10%), 237.05 (11%), 205.08 (8%), 186.07 (9%). HR-MS: [M^+^] 380.1060; [M^+^+1] 381.1094; [M^+^+3] 382.1015

*Ethyl 2-((3-(2-ethoxy-2-oxoethyl)-4-oxo-4,5-dihydro-3H-imidazo[4,5-c]quinolin-2-yl)thio)acetate (***5a***).* Product characterization: Colorless solid, melting point 200–204 °C (from ethyl acetate). %). EA: % calcd (found) for C_18_H_19_N_3_O_5_S (389.37): C 55.52 (55.68), H 4.92 (4.89), N 10.79 (10.75), S 8.24 (8.17). IR (KBr): 3453, 3165, 3126, 3052, 2986, 2846, 1738, 1662, 1556, 1466, 1436, 1376, 1304, 1224, 1174, 1098, 1025, 965, 898, 875, 762, 724, 701, 681, 669, 597, 512, 456 cm^−1^. ^1^H, ^13^ C{^1^H}, and ^15^N NMR data are referred in Table 2. EI-MS (*m*/*z*, %): 390.11 (10%), 362.08 (8%), 344.07 (25%), 316.4 (15%), 286.06 (37%), 258.03 (100%), 242.04 (40%), 230.04 (98%), 216.02 (12%), 198.07 (17%), 186.07 (40%). HR-MS: [M^+^] 390.1114; [M^+^ + 1] 391.1148; [M^+^ + 3] 392.1069.

*Ethyl 2-((3-(2-ethoxy-2-oxoethyl)-4-oxo-5-methyl-4,5-di-hydro-3H-imidazo[4,5-c]quinolin-2-yl)thio)- acetate (***5b***).* Product characterization: Colorless solid, melting point 129–132 °C (from benzene/hexane). EA: % calcd (found) for C_19_H_21_N_3_O_5_S (403.45): calcd C 56.57 (56.75), H 5.25 (5.33), N 10.42 (10.46), S 7.95 (7.86). IR (KBr): 3452, 3067, 2984, 2936, 2904, 1734, 1653, 1577, 1503, 1466, 1443, 1416, 1378, 1707, 1264, 1224, 1159, 1117, 1095, 1029, 980, 946, 898, 874, 859, 780, 755, 714, 677, 657, 573, 551, 480 cm^−1^. ^1^H, ^13^ C{^1^H}, and ^15^N NMR data are referred in Table 2. EI-MS (*m*/*z*, %): 404.13 (24%), 376.10 (15%), 358.08 (37%), 330.05 (17%), 300.08 (65%), 272.05 (100%), 256.05 (18%), 242.04 (61%), 230.04 (18%) 200.08 (16%). HR-MS: [M^+^] 404.1270; [M^+^ + 1] 405.1306; [M^+^ + 3] 406.1226.

*Ethyl 2-((3-(2-ethoxy-2-oxoethyl)-4-oxo-5-phenyl-4,5-di-hydro-3H-imidazo[4,5-c]quinolin-2-yl)thio)- acetate (***5c***).* Product characterization: Colorless solid, melting point 129–131 °C (from benzene/hexane). EA: % calcd (found) for C_24_H_23_N_3_O_5_S (465.46): C 61.93 (61.75), H 4.98 (5.17), N 9.03 (8.96), S 6.89 (6.79). IR (KBr): 3034, 2984, 2934, 1755, 1660, 1576, 1510, 1491m, 1462, 1440, 1417, 1376, 1310, 1261, 1219, 1200, 1175, 1148, 1127, 1096, 1024, 967, 896, 863, 847, 804, 767, 700, 679, 583, 531 cm^−1^. ^1^H, ^13^ C{^1^H}, and ^15^N NMR data are referred in Table 2. EI-MS (*m*/*z*, % 466.14 (22%), 438.11 (10%), 420.10 (24%), 393.07 (10%), 362.10 (48%), 334.06 (100%), 318.08 (10%), 306.07 (32%), 278.06 (14%), 262.10 (14%), 247.08 (8%). HR-MS: [M^+^] 466.1429; [M^+^ + 1] 467.1464; [M^+^ + 3] 468.1385.

*Ethyl 2-((1-(2-ethoxy-2-oxoethyl)-4-oxo-4,5-dihydro-1H-imidazo[4,5-c]chinolin-2-yl)thio)acetate (***6a***).* Product characterization: Colorless solid, melting point 235–237 °C (from ethanol). EA: % calcd (found) for C_18_H_19_N_3_O_5_S (389.43): C 55.52 (55.43), H 4.92 (4.84), N 10.79 (10.65), S 8.23 (8.07). IR (KBr): 3457, 3174, 3016, 2973, 2876, 1743, 1680, 1549, 1514, 1456, 1428, 1403, 1370, 1350, 1309, 1225, 1174, 1117, 1023, 977, 941, 903, 873, 802, 755, 725, 687, 670, 565, 514 cm^−1^. ^1^H, ^13^ C{^1^H}, and ^15^N NMR data are referred in Table 2. ESI-MS: 389 ([M+H]^+^, 412 [M+Na]^+^, 428 [M+K]^+^.

^1^H-NMR spectra of products are given in Figures S1–S5.

### 3.3. Analytical Methods

Melting points were determined on a Kofler block (Vienna, Austria).

IR (KBr) spectra were recorded on a Smart OMNI-Transmission Nιcolet iS10 spectrophotometer (Waltham, MA, USA).

The ^1^H, ^13^C{^1^H}, and ^15^N NMR spectra were recorded on a Bruker Avance III HD 500 spectrometer (500.13 MHz for ^1^H, 125.76 MHz for ^13^C{^1^H}, and 50.68 MHz for ^15^N) in DMSO-*d*_6_. ^1^H and ^13^C chemical shifts are given on the δ scale (ppm) and are referenced to internal TMS (δ = 0.0). ^15^N chemical shifts were referred to external neat CH_3_NO_2_ in a co-axial capillary (δ = 0.0). All 2D experiments (gradient-selected (gs)-COSY, gs-TOCSY, gs-NOESY, gs-HMQC, gs-HMQC-TOCSY, gs-HMQC-RELAY, and gs-HMBC) were performed using manufacturer’s software (TOPSPIN 3.5, Rheinstetten, Germany).

The electrospray mass spectra (ESI-MS) were recorded using an amaZon X ion-trap mass spectrometer (Bruker Daltonics, Bremen, Germany) equipped with an electrospray ion source. All experiments were conducted in both positive and negative polarity mode. Individual samples (with a concentration of 500 ng·mL^−1^) were infused into the ESI source as methanol/water (1:1, *v*/*v*) solutions via a syringe pump with a constant flow rate of 3 μL·min^−1^. The other instrumental conditions were as follows: Electrospray voltage of ±4.2 kV, capillary exit voltage of ±140 V, drying gas temperature The GC-MS analyses were measured with Shimadzu GC-MS QP-2010 with quadrupole mass detector and column EQUITY 1 (30 m, 0.32 mm, 1 μm). Temperature program: 100 °C/7 min, 30 °C/min, temperature of spray 250 °C. All experiments were carried out at constant linear rate of 52 cm/s. HR-MS spectra were recorded by micrOTOF-QII (Agilent Technologies, Santa Clara, CA, USA), and calibration was performed with TuneMIX (Agilent Technologies, Santa Clara, CA, USA). Full-mass range 50–750 *m*/*z*. Full mass resolution 140,000. MS/MS parameters: Type of HCD fragmentation (high energy collision dissociation), CE (collision energy) 35. Spray voltage positive ion mode 4100 V. Sheath gas 10 arbitrary units. Auxiliary gas 0 arbitrary units. Ion transfer temperature 320 °C. Vaporisation temperature 250 °C.

Column chromatography was carried out on silica gel (Merck, grade 60, 70–230 mesh) using successive mixtures of chloroform/ethanol (in ratios from 99:1 to 8:2) (Figure S1) or benzene/ethyl acetate (in ratios from 99:1 to 8:2) (Figure S2).

Reactions as well as the course of separation and also the purity of all substances were monitored by TLC (elution systems benzene/ethyl acetate (4:1) (Figure S3), chloroform/ethanol (9:1 and 1:1) (Figures S4 and S5), and chloroform/ethyl acetate (7:3) (Figure S6) on Alugram^®^ SIL G/UV_254_ foils (Macherey-Nagel, (Dueren, Germany).

Elemental analyses (C, H, N, S) of the obtained compounds were performed with an analyser Vario EL III C, H, N from Elementar Company (Langenselbold, Germany).

The thermogravimetric analysis of mono- and diesters was performed in the ceramic crucible using microthermogravimeter Mettler Toledo 851e Prague, Czech republic). The measurement conditions were as follows: Sample weight: 7–8 mg, temperature range: 20–600 °C, atmosphere: Argon, heating rate 10 °C/min.

Thermal measurements were carried out using the standard differential scanning calorimetry (DSC) a TA 2920 from TA Instruments, Inc. (New Castle, DE, USA). This calorimeter is the heat-flux type and use a mechanical refrigerator to cool the sample. All experiments were performed in a nitrogen atmosphere with a constant flow rate of around 50 mL/min [24]. The series of experimental heat flow rates were obtained by standard DSC at a heating rate of 10 deg/min after previous cooling also at 10 deg/min. The temperature and heat flow rate calibration in the DSC apparatus was performed using parameters of melting indium [T_m_(onset) = 156.6 °C, ΔH_f_ = 28.45 J·g (3.281 kJ/mol)], [24]. The masses of the samples used for the DSC measurements were 10–30 mg. The thermal data were collected from the first and second heating run after controlled cooling. By the standard DSC measurements, total heat flow rate was obtained for investigated samples.

The molecular modelling was performed using a set of computational methods based on the electron density of the tested compounds in the stationary state, i.e., density functional theory DFT [25], with use the Gaussian 09 application [26]. The B3LYP functional was selected as the most suitable for the tasks associated with organic compounds. The quantum-mechanical calculations were performed using the 6-311++g(d,p) basis set [27], with Grimme dispersion correction (D3 version) which considered intramolecular hydrogen bonds and electrostatic interactions [28]. The charge value was 0 (zero) and spin state value was 1, because the calculated models of molecules were neutral, in single state. The values of the mole percentage were calculated on the basis of Gibbs free energy (enthalpy). Gibbs free energy was calculated at temperature 298.15 K. Frequency calculations were performed at the same level of theory as the geometry optimization to confirm whether the obtained structures were minima (no imaginary frequency) or transition states (only one imaginary frequency). Structure visualization was performed using GaussView [29].

## 4. Conclusions

In conclusion, it should be emphasized that the described method enables the preparation, with good to very good yields, of new mono- and diesters with imidazoquinolinone ring, starting from 2,3-dihydro-2-thioxo-1*H*-imidazo[4,5-*c*]quinolin-4(5*H*)-ones.

These results also increase the variety of methods for the preparation of diesters with heterocyclic rings. All the prepared compounds have not been described previously in the literature.

The structure of monoesters is different than it results from the structure of the raw materials. Substrates have the confirmed tautomeric structure—keto which is the most energetically stable. Enol (thiol) form of 2,3-dihydro-2-thioxo-1H-imidazo[4,5-c]quinolin-4(5H)-ones was identified in the mono- and next in diester structure. Enolization process takes place during substitution reaction. In this way, the most stable structure of mono- and diester are formed. It was proved and confirmed by molecular modeling that enolization process occurs only with participation nitrogen atom number 1.

Substitution of the second ester group takes place at nitrogen atom number 3 despite of the fact that in the case of some used 2,3-dihydro-2-thioxo-1H-imidazo[4,5-c]quinolin-4(5H)-ones is the possibility of reaction at nitrogen atom number 5. Nevertheless, such reaction is not energetically beneficial.

The obtained diesters have quite good thermal stability, which enables their use in the synthesis of thermally stable polymers.

Furthermore, both the mono- and diester have potential biological activity. It will be the subject of the next separated manuscript.

## Figures and Tables

**Table 1:** Results of the reaction of **2** with ethyl bromoacetate (EBA).

Starting Compound	Molar Ratio of 2: EBA	Yield of 4 (%)	Yield of 5 (%)	Yield of 6a (%)
**2a** 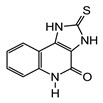	1:1.1	6	28	5
	1:2.4	5	43	*7*
	1:4	4	45	0
**2b** 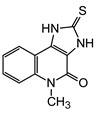	1:1.1	49	16	0
	1:2.4	74	1	0
	1:4	0	79	0
**2c** 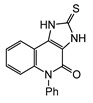	1:1.1	83	4	0
	1:2.4	56	27	0
	1:4	0	80	0

**Table 2:** ^1^H, ^13^C{^1^H} and ^15^N chemical shifts and ^1^*J*(^15^N, H) (Hz, ± 0.3 Hz) of compounds **4a**–**c**, **5a**–**c**, and **6a** in DMSO.

Position	4a		4b		4c		5a		5b		5c		6a	
	δ(H)	δ(C)	δ(H)	δ(C)	δ(H)	δ(C)	δ(H)	δ(C)	δ(H)	δ(C)	δ(H)	δ(C)	δ(H)	δ(C)
1 N	-	n.o. ^a^	-	n.o. ^a^	-	n.o. ^a^	-	n.o. ^a^	-	n.o. ^a^	-	n.o. ^a^	-	−224.1 ^a^
2 CS	-	149.0	-	148.8	-	149.8	-	151.1	-	151.4	-	151.9	-	148.4
3 N	-	n.o. ^a,b^	-	n.o. ^a,d^	-	n.o.^a, e^	-	−225.5 ^a^	-	−225.7 ^a^	-	224.8 ^a^	-	n.o. ^a^
3a	-	122.2	-	122.3	-	121.8	-	121.8	-	121.2	-	121.3	-	131.6
4 C=O	-	154.4	-	154.3	-	153.9	-	154.7	-	154.1	-	154.2	-	156.5
5 N-R	-	−235.7 ^a^	-	−242.4 ^a^	-	−220.7 ^b^	-	−234.8 ^a^	-	−241.4 ^a^	-	−220.1 ^a^	-	−233.8 ^a^
5a	-	136.3	-	137.1	-	138.0	-	136.2	-	137.1	-	137.2	-	136.6
6	7.42	116.0	7.55	115.7	6.55	116.4	7.42	116.1	7.57	116.8	6.58	116.5	7.45	116.5
7	7.42	128.0	7.53	128.4	7.32	122.4	7.42	122.3	7.57	128.8	7.40	128.5	7.45	128.4
8	7.22	121.9	7.33	122.2	7.28	128.0	7.28	128.4	7.36	122.8	7.37	122.8	7.21	121.9
9	7.94	121.4	8.03	121.8	8.07	121.8	7.99	121.3	8.06	121.7	8.13	121.7	7.78	120.6
9a	-	115.8	-	115.7	-	116.1	-	115.3	-	115.9	-	115.8	-	111.2
9b	-	144.5	-	142.6	-	143.9	-	144.0	-	142.8	-	143.6	-	135.8
SCH_2_	4.22	33.7	4.23	33.7	4.26	33.7	4.22	34.7	4.24	34.6	4.32	34.7	4.19	35.1
COO	-	168.6	-	168.6	-	168.6	-	168.3	-	168.3	-	168.2	-	168.3
OCH_2_	4.02	61.2	4.11	61.2	4.14	61.2	4.12	61.3	4.14	61.3	4.16	61.6	4.13	61.4
CH_3_	1.16	14.0	1.17	14.0	1.18	14.1	1.17	14.0	1.19	14.0	1.23	14.0	1.18	14.0
NCH_2_	-	-	-	-	-	-	5.33	46.4	5.31	46.4	5.32	46.4	5.50	47.8
COO	-	-	-	-	-	-	-	167.3	-	167.4	-	167.2	-	167.3
OCH_2_	-	-	-	-	-	-	4.17	61.6	4.18	61.6	4.16	61.3	4.22	61.9
CH_3_	-	-	-	-	-	-	1.22	14.0	1.22	14.00	1.23	14.0	1.20	14.0
1′(R)	11.60	88.0 ^c^	3.69	29.0	-	138.5	11.76	88.8 ^c^	3.64	28.7	-	138.5	11.71	89.2 ^c^
2’(R)			-	-	7.34	129.6	-	-	-	-	7.34	129.4	-	-
3´(R)			-	-	7.64	130.0	-	-	-	-	7.64	130.0	-	-
4´(R)			-	-	7.56	128.7	-	-	-	-	7.57	128.9	-	-

^a^ δ(^15^N); ^b^ δ(NH) = 13.67; ^c 1^*J*(^15^N, H); ^d^ δ(NH) = 13.67; ^e.^δ(NH) = 13.82.

**Table 3:** Possible tautomeric forms of compounds **2a, 2b, 2c.**

CompoundForm	2a: R = H	2b: R = Me	2c: R = Ph
keto	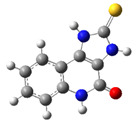	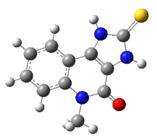	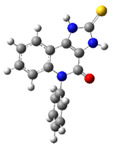
enol N^(1)^-SH	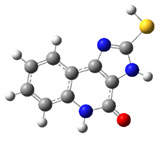	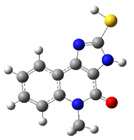	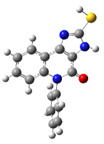
enol N^(3)^-SH	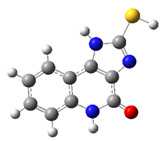	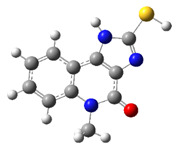	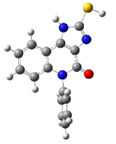
enol N^(5)^-OH	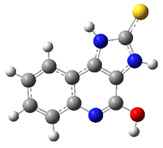	not possible	not possible
enol N^(1)^-SH,N^(5)^-OH	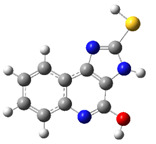	not possible	not possible
enol N^(3)^-SH,N^(5)^-OH	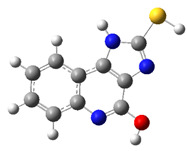	not possible	not possible

**Table 4:** Results of the analysis of conformer population of compounds **2.**

Compound		Total Energy, kJ/mol	Percentage, mol%	
**2a**	keto	−2686131.32	99.9976	100
	enol N^(1)^-SH	−2686028.28	0.0019	
	enol N^(3)^-SH	−2686104.43	0.0000	
	enol N^(5)^-OH	−2686066.23	0.0004	
	enol N^(1)^-SH,N^(5)^-OH	−2686083.71	0.0000	
	enol N^(3)^-SH,N^(5)^-OH	−2686057.23	0.0000	
**2b**	keto	−2789294.72	99.9987	100
	enol N^(1)^-SH	−2789266.88	0.0013	
	enol N^(3)^-SH	−2789244.40	0.0000	
**2c**	keto	−3292711.66	99.9989	100
	enol N^(1)^-SH	−3292683.43	0.0011	
	enol N^(3)^-SH	−3292661.65	0.0000	

**Table 5:** HOMO electron densities at selected atoms.

Compound		HOMO Electron Densities at Selected Atoms in Molecule [%]				
		N^(1)^	N^(3)^	N^(5)^	S	O
**2a**	keto	5	10	0	51	5
**2b**		5	0	-	51	5
**2c**		5	0	-	50	5
**2a**	enol N^(1)^-SH	-	4	5	5	16
**2b**		-	4	-	4	17
**2c**		-	4	-	4	16

**Table 6:** Possible conformers of products of reaction of compounds 2 with ethyl bromoacetate—monoderivatives.

Compound	Place of Substitution	Total Energy, kJ/mol	Percentage, mol%			Conformer Symbol
**4a**	S	−3387684.80	76.32	80.66	100	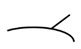
		−3387676.06	2.25			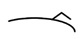
		−3387674.21	1.06			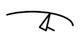
		−3387674.14	1.03			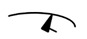
	N^(1)^	−3387678.85	6.91	13.94		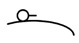
		−3387678.89	7.03			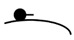
	N^(3)^	−3387675.59	1.85	3.73		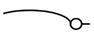
		−3387675.62	1.88			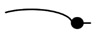
	N^(5)^	−3387673.62	0.84	1.67		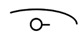
		−3387673.61	0.83			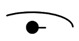
**4b**	S	−3490846.85	69.59	73.93	100	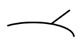
		−3490838.19	2.11			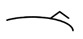
		−3490836.69	1.16			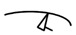
		−3490836.49	1.07			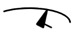
	N^(1)^	−3490842.13	10.40	20.63		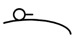
		−3490842.09	10.23			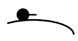
	N^(3)^	−3490838.81	2.72	5.44		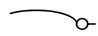
		−3490838.81	2.72			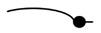
**4c**	S	−3994263.56	63.39	67.50	100	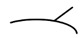
		−3994255.02	2.03			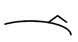
		−3994253.55	1.12			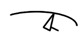
		−3994253.19	0.97			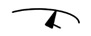
	N^(1)^	−3994258.94	9.85	17.96		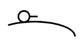
		−3994258.46	8.11			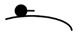
	N^(3)^	−3994258.17	7.22	14.55		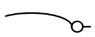
		−3994258.21	7.33			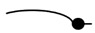

**Table 7:** Possible conformers of products of the reaction of compounds 2 with ethyl bromoacetate—bisderivatives.

Compound	Place of Substitution	Total Energy, kJ/mol	Percentage, mol%			Conformer Symbol
**5a**	S, N^(3)^	−4089228.82	30.97	59.15	100	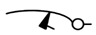
	S, N^(3)^	−4089229.05	28.18			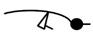
	S, N^(3)^	−4089226.63	11.47	22.93		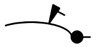
	S, N^(3)^	−4089226.59	11.47			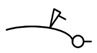
	S, N^(3)^	−4089224.39	4.81	9.54		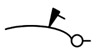
	S, N^(3)^	−4089224.44	4.73			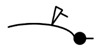
	S, N^(3)^	−4089224.09	4.17	8.37		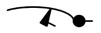
	S, N^(3)^	−4089224.08	4.19			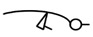
**6a**	S, N^(1)^	(*)	0.02	0.04		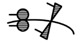
	S, N^(1)^	(**)	0.02			
**5b**	S, N^(3)^	−4192391.75	36.43	68.44	100	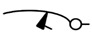
	S, N^(3)^	−4192392.07	32.01			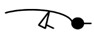
	S, N^(3)^	−4192388.72	8.75	18.19		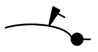
	S, N^(3)^	−4192388.54	9.44			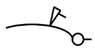
	S, N^(3)^	−4192386.53	3.78	7.68		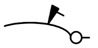
	S, N^(3)^	−4192386.46	3.90			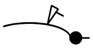
	S, N^(3)^	−4192385.66	2.93	5.68		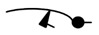
	S, N^(3)^	−4192385.83	2.74			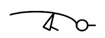
**6b**	S, N^(1)^	(*)	0.01	0.02		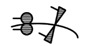
	S, N^(1)^	(**)	0.01			
**5c**	S, N^(3)^	−1788.538796	32.54	62.24	100	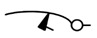
	S, N^(3)^	−1788.538946	29.70			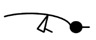
	S, N^(3)^	−1788.537784	9.62	19.13		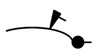
	S, N^(3)^	−1788.537796	9.50			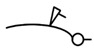
	S, N^(3)^	−1788.53719	4.95	10.02		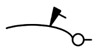
	S, N^(3)^	−1788.537169	5.06			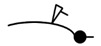
	S, N^(3)^	−1788.537049	4.25	8.61		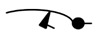
	S, N^(3)^	−1788.537024	4.36			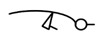
**6c**	S, N^(1)^	(*)	0.01	0.02		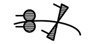
	S, N^(1)^	(**)	0.01			

(*) Group of five isomers and their mirror images (**).

**Table 8:** Schematism of the conformer symbol.

Possibilities	Sample Structure and Explanations	Ideogram
Place of substitution:		
(a) N^(1)^ (b) S (c) N^(3)^	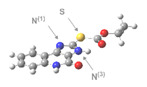	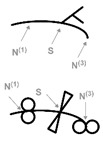
Shape of substituent’s chain (only in plane)		
(a) line (b) polyline	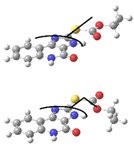	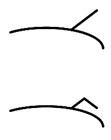
Shape filling		
(a) solid = over the plane of the rings (b) clear = below the plane of the rings	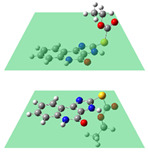	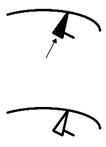
Substituent plane inclination		
(a) right (b) left (seen from the center of the rings)	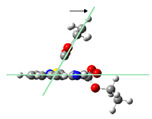	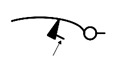
Substituent’s –OC_2_H_5_ chain position		
(a) over rings (b) outside of the rings	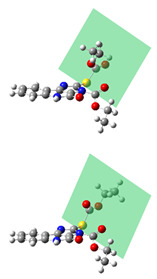	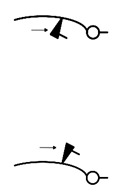

## Supplementary Materials

The following are available online, Figure S1: ^1^H-NMR spectrum of ethyl 2-((4-oxo-4,5-dihydro-3H-imidazo[4,5-c]quinolin-2-yl)thio)acetate (4a),Figure S2: ^1^H-NMR spectrum of ethyl 2-((4-oxo-5-methyl-4,5-dihydro-3H-imidazo[4,5-c]-quinolin-2-yl)thio)acetate (4b), Figure S3: ^1^H-NMR spectrum of ethyl 2-((4-oxo-5-phenyl-4,5-dihydro-3H-imidazo[4,5-c]-quinolin-2-yl)thio)acetate (4c), Figure S4: ^1^H-NMR spectrum of ethyl 2-((3-(2-ethoxy-2-oxoethyl)-4-oxo-4,5-dihydro-3H-imidazo[4,5-c]quinolin-2-yl)thio)acetate (5a), Figure S5: ^1^H-NMR spectrum of ethyl 2-((3-(2-ethoxy-2-oxoethyl)-4-oxo-5-methyl-4,5-di-hydro-3H-imidazo[4,5-c]quinolin-2-yl)thio)- acetate (5b), Figure S6: ^1^H-NMR spectrum of ethyl 2-((3-(2-ethoxy-2-oxoethyl)-4-oxo-5-methyl-4,5-di-hydro-3H-imidazo[4,5-c]quinolin-2-yl)thio)- acetate (5c). Table S1: Possible conformers of compounds 4, Table S2: Possible conformers of compounds 5.
