# Supplementary material for: New Mono- and Diesters with Imidazoquinolinone Ring- Synthesis, Structure Characterization, and Molecular Modeling
Source: Molecules. 2020 Sep 19;25(18):4303. doi: 10.3390/molecules25184303 (PMC7571163; doi:10.3390/molecules25184303)
Supplement: Supplementary file 1 [file molecules-25-04303-s001.pdf]

New Mono- and Diesters With Imidazoquinolinone Ring - Synthesis, Structure Characterization and Molecular Modeling

Iwona Zarzyka [1,*], Antonin Klásek [2], Karol Hęclik [1], Antonin Lyčka [3], Radek Bartošík [2], and Lucjan Dobrowolski, [1]

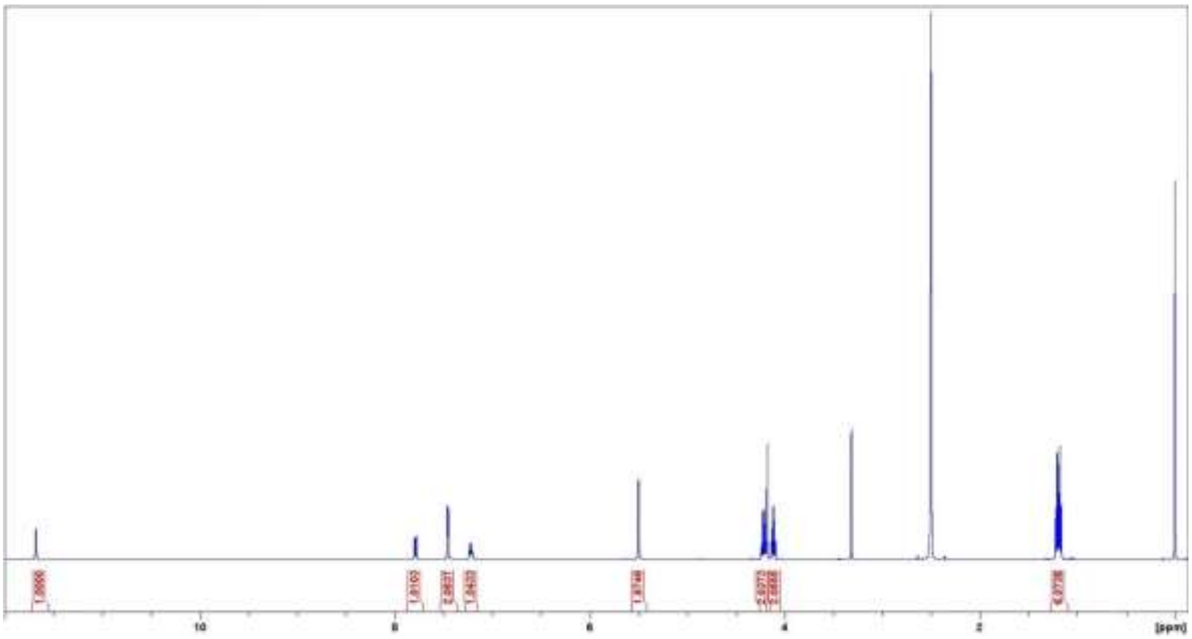

Fig. 1S. ^{1}H-NMR spectrum of ethyl 2-((4-oxo-4,5-dihydro-3H-imidazo[4,5-c]quinolin-2-yl)thio)acetate (4a)

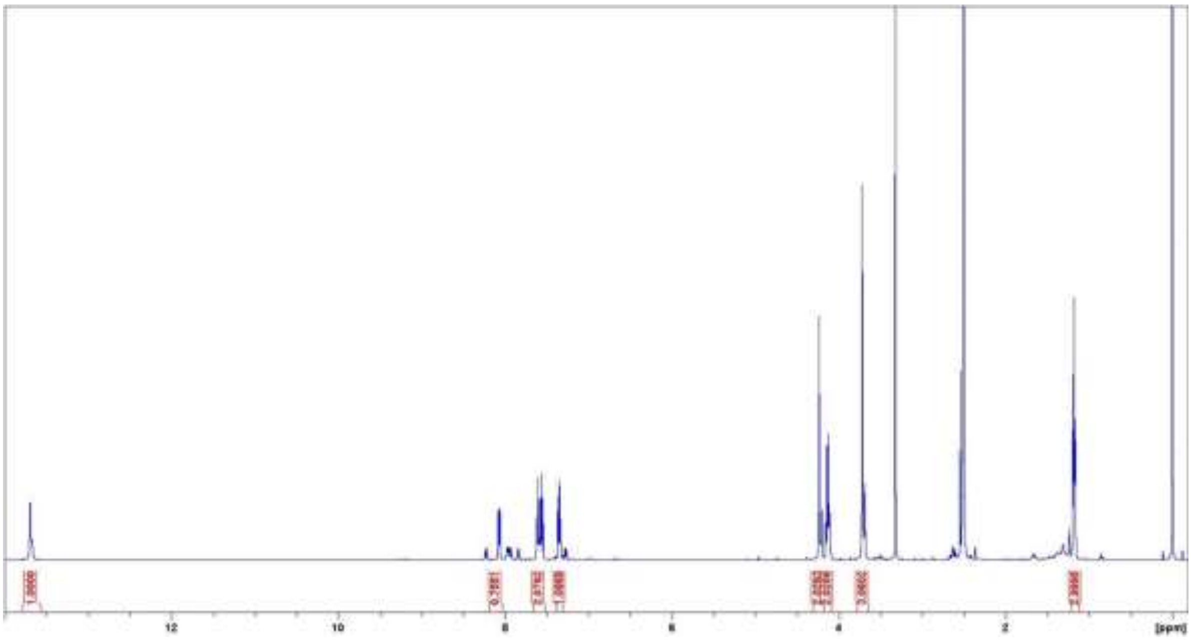

Fig. 2S. ^{1}H-NMR spectrum of ethyl 2-((4-oxo-5-methyl-4,5-dihydro-3H-imidazo[4,5-c]-quinolin-2-yl)thio)acetate (4b).

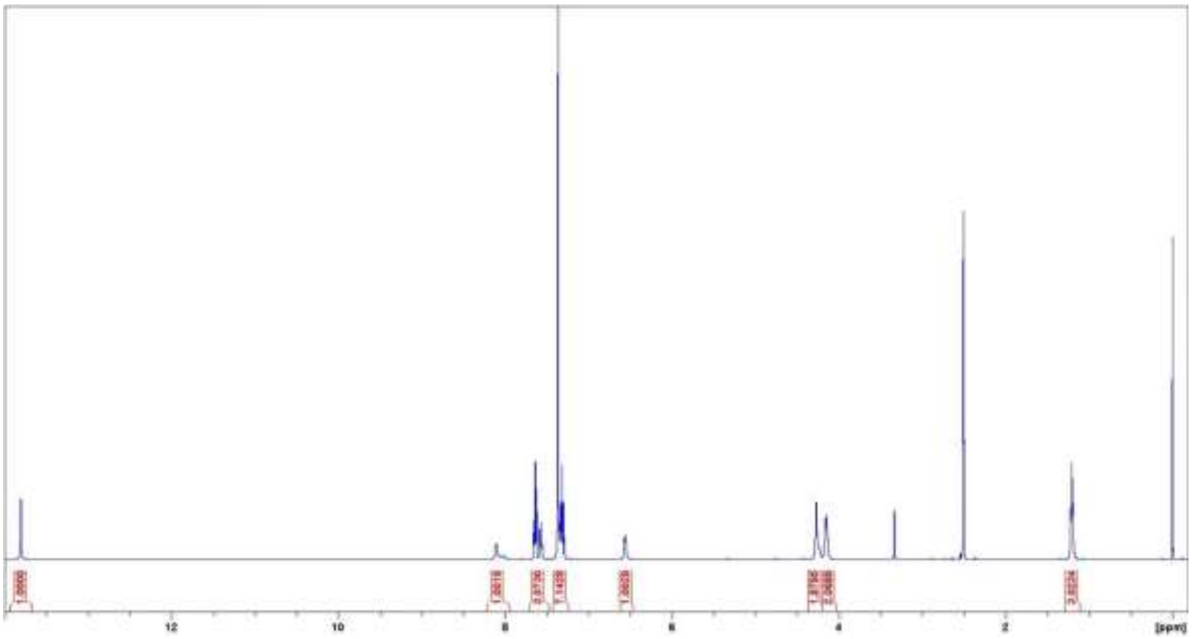

Fig. 3S. ^{1}H-NMR spectrum of ethyl 2-((4-oxo-5-phenyl-4,5-dihydro-3H-imidazo[4,5-c]-quinolin-2-yl)thio)acetate (4c).

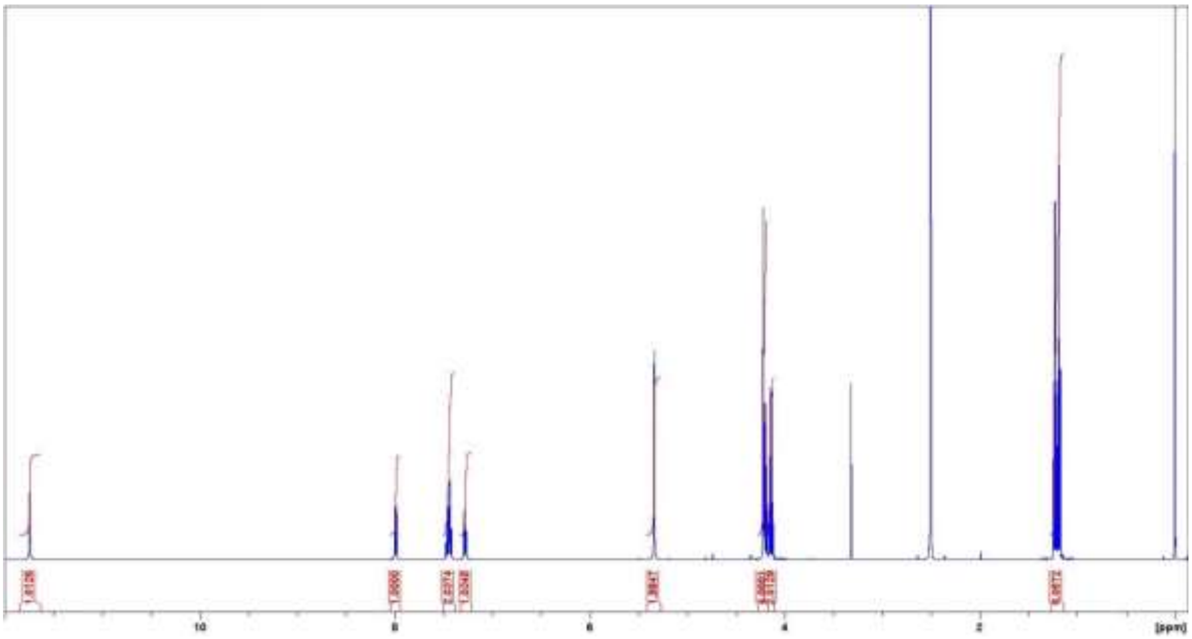

Fig. 4S. ^{1}H-NMR spectrum of ethyl 2-((3-(2-ethoxy-2-oxoethyl)-4-oxo-4,5-dihydro-3H-imidazo[4,5-c]quinolin-2-yl)thio)acetate (5a).

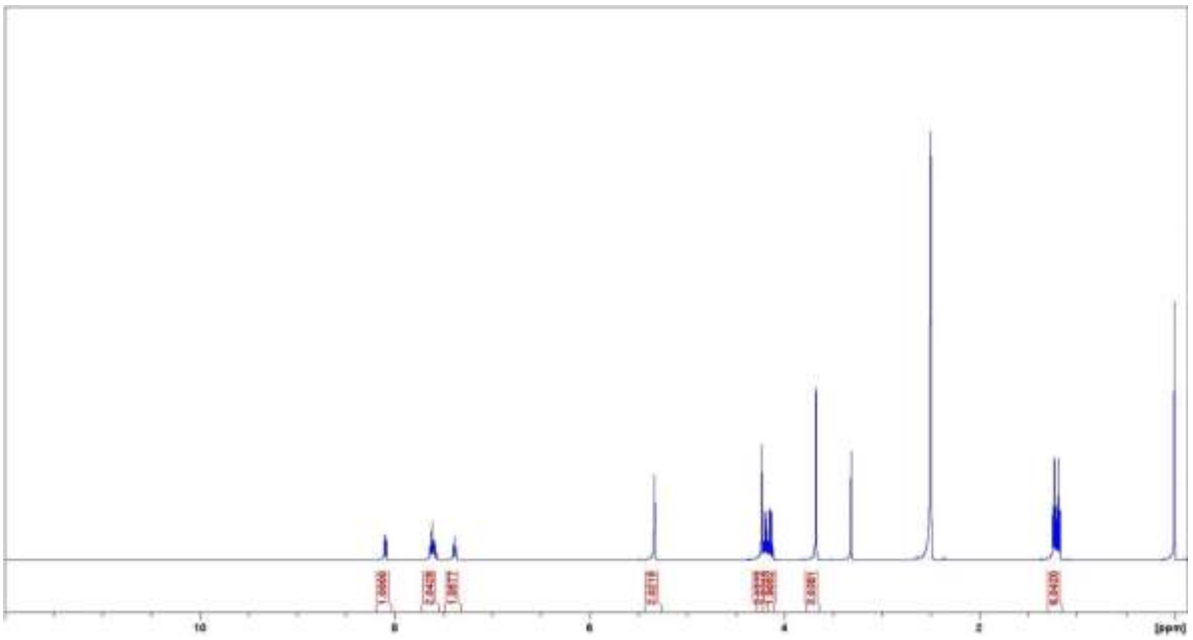

Fig. 5S. ^{1}H-NMR spectrum of ethyl 2-((3-(2-ethoxy-2-oxoethyl)-4-oxo-5-methyl-4,5-di-hydro-3H-imidazo[4,5-c]quinolin-2-yl)thio)- acetate (5b).

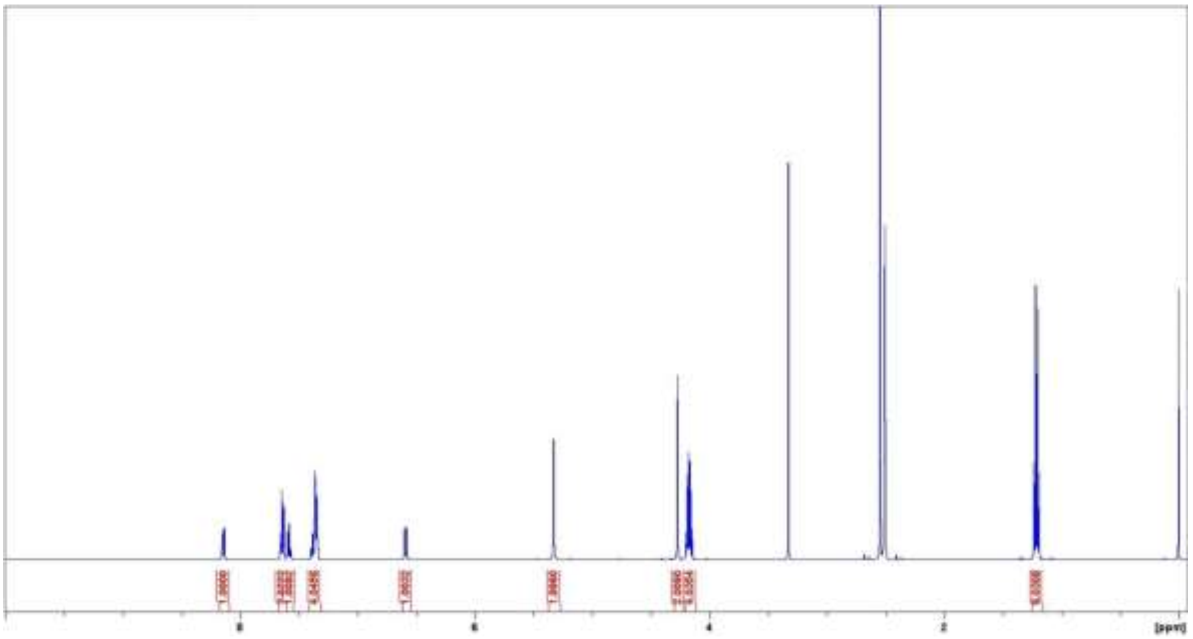

Fig. 6S. ^{1}H-NMR spectrum of ethyl 2-((3-(2-ethoxy-2-oxoethyl)-4-oxo-5-methyl-4,5-di-hydro-3H-imidazo[4,5-c]quinolin-2-yl)thio)- acetate (5c).

Table 1S. Possible conformers of compounds **4**

Compound	Conformer symbol	Conformer structure
	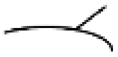	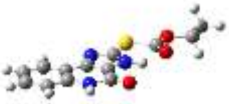
	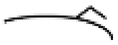	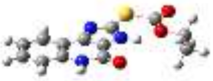
	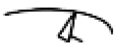	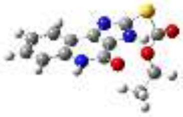
	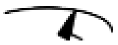	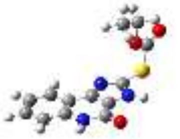
	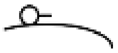	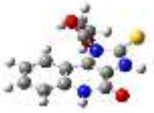
**4a**	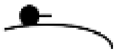	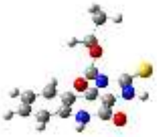
	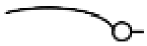	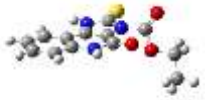
	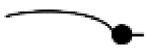	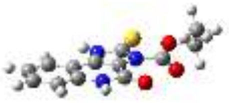
	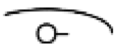	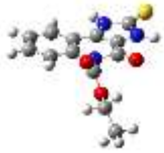
	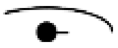	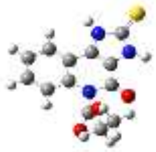

	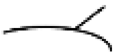	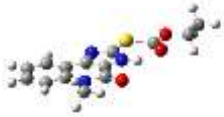
	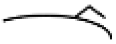	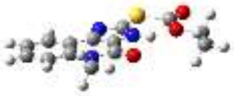
	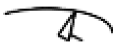	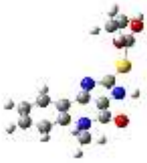
	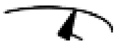	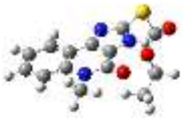
**4b**	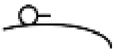	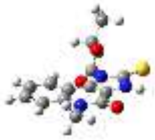
	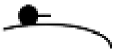	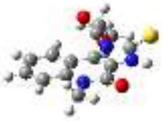
	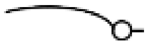	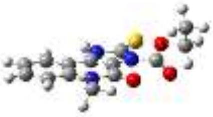
	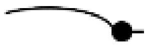	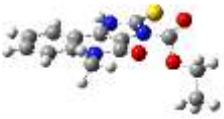
	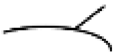	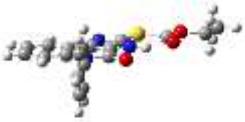
**4c**	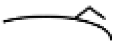	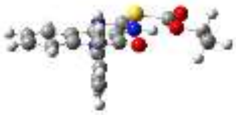
	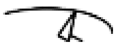	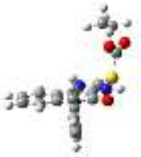

	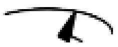	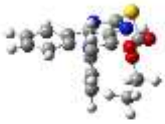
	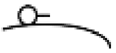	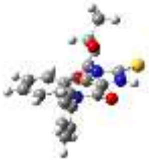
	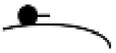	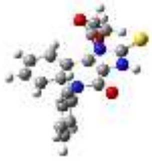
	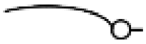	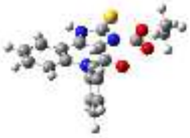
	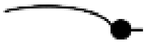	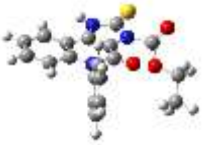

Table 2S. Possible conformers of compounds **5**

**Compound**	**Conformer symbol**	**Conformer structure**
**5a**	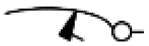	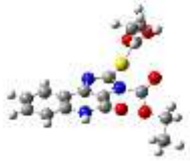
	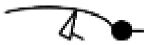	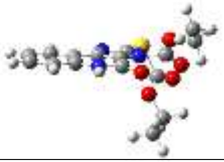
	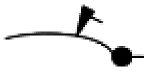	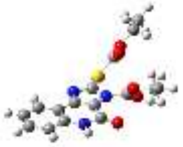
	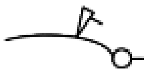	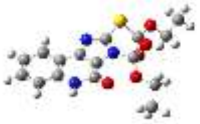
	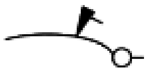	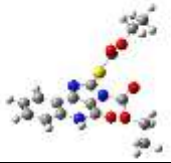
	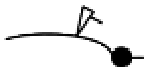	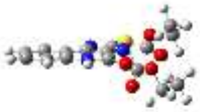
	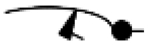	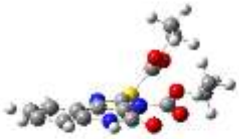
	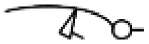	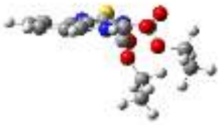
**5b**	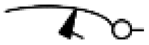	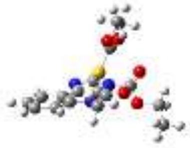
	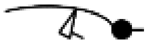	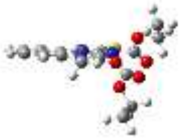

	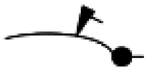	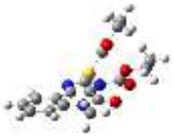
	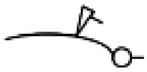	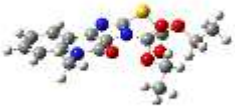
	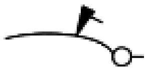	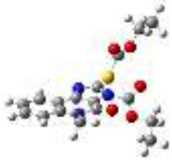
	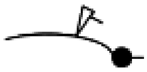	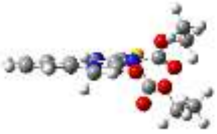
	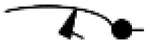	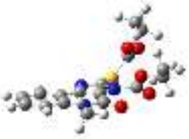
	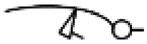	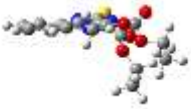
	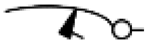	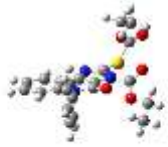
	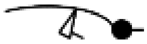	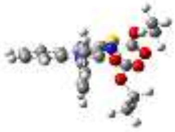
**5c**	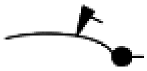	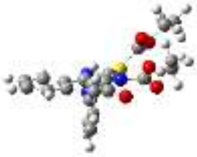
	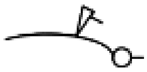	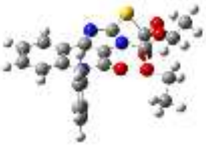
	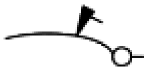	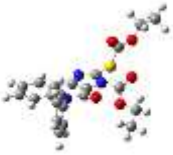

	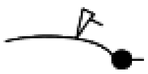	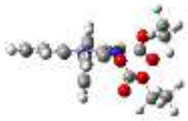
	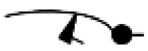	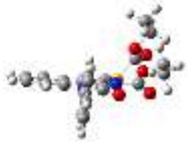
	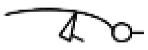	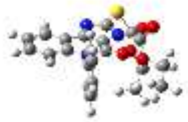
